# Consequences of grouped data for testing for departure from circular uniformity

**DOI:** 10.1007/s00265-017-2393-2

**Published:** 2017-10-28

**Authors:** Rosalind K. Humphreys, Graeme D. Ruxton

**Affiliations:** School of Biology, University of St Andrews, Dyer’s Brae House, St Andrews, KY16 9TH UK

**Keywords:** Chi-squared test, Kolmogorov-Smirnov test, Ordered categories, Periodic data, Rayleigh test

## Abstract

Limits to the precision of circular data often cause grouping of data points into discrete categories, but the effects of grouping on tests for circular uniformity have been little explored. The Rayleigh test is often applied to grouped circular data, despite it being designed for continuous data and the statistical literature recommending a suite of alternative tests specifically designed for grouped data. Here, we investigated the performance of the Rayleigh test relative to four alternatives for testing the null hypothesis of uniformity in grouped circular data. We employed simulation, grouping data into a discrete number of same-sized categories and with samples drawn from a range of different distributions. We found that grouping had little effect on the type I error rate or the power of the Rayleigh test, and that the power of the Rayleigh test was very similar to that of the previously recommended alternative tests designed specifically for grouped circular data. It may thus be appropriate to apply the Rayleigh test to grouped data, provided the situation is one in which the test has substantial statistical power.

**Electronic supplementary material:**

The online version of this article (10.1007/s00265-017-2393-2) contains supplementary material, which is available to authorized users.

## Introduction

Circular data is characterised by an inherent periodicity absent from measurements made on a linear scale (such as mass or length). Such data is generated by a range of common measurements across scientific disciplines. The most obvious situation involves angles, compass bearings or orientations. However, also common are measurements over time where an inherent periodicity is relevant: e.g. time of day, seasonality and point in the lunar cycle. Recent articles in *Behavioral Ecology and Sociology* (BES) have used circular statistics to explore issues as diverse as daily variation in mammalian activity (Fancourt 2016), orientation of sandhoppers with respect to the moon as part of their navigation (Ugolini 2016), search strategies of desert ants (Schultheiss et al. 2016) and the directions with respect to a threat adopted by fleeing deer (Obleser et al. 2016). In the first 6 months of 2017, BES published four papers utilising circular statistics investigating the relative positioning of individuals in an orb-weaving spider colony (Yip et al. 2017), navigation in ants (Amador-Vargas and Mueller 2017), avian navigation by the stars (Pakhomov et al. 2017) and integration of magnetic and visual navigational cues in shorebirds (Vanni et al. 2017).

Circular data needs special treatment in data analysis, and a range of texts has been dedicated to describing this: e.g. Batschelet 1981; Fisher 1995; Mardia and Jupp 2000; Jammalamadaka and SenGupta 2001; Pewsey et al. 2013; Ley and Verdebout 2017. The most common statistical exploration of circular data involves testing to see if there is a bias in the distribution around the circle or whether the null hypothesis that the underlying population involves a uniform spread around the circle is supported. For example, one might test whether the compass directions of the initial flights of released homing pigeons are random or show a bias. In this situation, one might expect that a bias would exist towards the direction of the home lofts of the pigeons. However, a low *p* value in a Rayleigh test would only provide support for rejection of the null hypothesis of a lack of bias leading to a uniform spread of directions around the circle. Exploration of the nature of any suggested bias might be performed by visual inspection and/or calculation of (for example) the mean direction. Provided the number of groups is at least eight, texts such as Jammalamadaka and SenGupta (2001) and Pewsey et al. (2013) recommend that summary statistics (and confidence intervals) be calculated making the assumption that each data point in a group takes the value of the midpoint of the relevant segment of the circle (see Pewsey et al. 2013 for details). For very course grouping into fewer than eight segments, Pewsey et al. (2013) present an alternate approach by Mardia (1972).

Just like linear measurements, circular measurements can be continuous or discrete. We surveyed recent papers in the top 20 ecological journals (based on citation rates) by searching for papers including the term ‘circular’ or ‘Rayleigh test’ and investigating what type of data the test was used on and how precisely the data measurements were taken in the most recent 60 relevant papers. We found that discrete data is commonplace, generally with the circular data being divided into *k* equally spaced ordered categories, with values of *k* = 4 (e.g. N, E, S or W), 8 (e.g. N, NE, … NW) or 12 (e.g. Jan, Feb, … Dec). Orientation or heading angles—of up to 360°—are sometimes split into eight sectors of 45° (Chittka et al. 1999; Davoren et al. 2003). Where orientation is relative to a structure or location, an angle of 180° may indicate an individual facing in the opposite direction, and orientation can be estimated to the nearest category, such as 12 sections of 15° (McLaughlin 2001). Twelve categories are also a common grouping for months of the year (Proença et al. 2012; Hirsch et al. 2016), but directional preferences and headings are commonly grouped by a greater number of equally spaced categories (Wiltschko et al. 2003, 2006, 2009; Kullberg et al. 2007; Winklhofer et al. 2013; Wystrach et al. 2015). There is no reason to expect that grouping in circular data would not also be commonplace in other fields. In recent BES papers, we found that it was not always possible to identify precision of measurements from the provided information, but we found examples of discrete data, with the circle being divided into equal-sized arcs numbering 48 (Fuchikawa et al. 2014), 36 (Obleser et al. 2016) and 8 (Villarreal and Gilbert 2014).

Our literature review also found a strong discrepancy between how the statistical literature recommends testing the null hypothesis of circular uniformity and the practice in empirical science. We found that in the ecological literature, circular uniformity is almost invariably tested using the Rayleigh test (introduced by Lord Rayleigh in 1880, but also defined in the monographs listed above). This is also true of recent BES papers (see references previously given). The Rayleigh test was designed to be applied to continuous data, and its application to discrete grouped data does not have a theoretical or empirical basis in the statistical literature. Instead, circular statistic texts recommend a suite of tests designed specifically for grouped circular data. Tests for unordered categories such as the chi-squared or *G* tests could be applied but, because these tests essentially leave unused information about the ordering of categories, they are not as powerful as purpose-designed tests for circular grouped data (Reijneveld 1990; Steele and Chaseling 2006; Watkins and Di Stefano 2013).

The most recent textbooks on circular statistics (Mardia and Jupp 2000; Pewsey et al. 2013) recommend three alternative tests for deviation from uniformity in grouped circular data: a modified Watson test by Choulakian et al. (1994), another modification of this test by Brown (1994) and a modification of the Kolmogorov-Smirnov test by Freedman (1979). Fisher (1995) recommends the first and the last of these tests. None of these texts offers advice on selecting one of these alternatives over another. The earlier text of Batschelet (1981) recommends the chi-squared test or the Rayleigh test only after a correction factor for grouping has been applied. Here, we explore the performance of the unmodified Rayleigh test in comparison to these four alternatives for testing the null hypothesis of uniformity in grouped circular data. BES authors regularly generate circular data, often in discrete rather than continuous form, and the Rayleigh test is the most commonly used statistical treatment of that data. Thus, this work should significantly enhance the statistical treatment of such data in the future and allow readers to draw more reliable inferences from previously published papers.

## Methods

For our simulations, samples were drawn from five different distributions: (i) a uniform distribution to allow us to study the type I error rate, (ii) a von Mises distribution (a symmetrical distribution often described as the circular equivalent to a normal distribution), (iii) a wrapped Cauchy distribution (another symmetrical unimodal distribution), (iv) an equal mixture of two von Mises distributions with means selected independently from a uniform distribution (giving a bimodal distribution or asymmetric unimodal one), and (v) an equal mixture of two von Mises distributions with mean values at the exactly opposite sides of the circle. For odd sample sizes, in the two mixture models, one subsample (selected stochastically) was one larger than the other. For the von Mises distribution, the concentration parameter *κ* was set to 0.8 throughout; for the wrapped Cauchy distribution, the concentration parameter *ρ* was set to 0.5. Descriptions of the uniform, von Mises and wrapped Cauchy distributions can be found in Pewsey et al. (2013). Samples were drawn stochastically using functions provided by the package *circular* (Agostinelli and Lund 2013) in R (R Core Team 2016). When data were grouped into *k* equal-length categories, each data point was rounded down to the nearest value in the list: 0, 2*π*/*k*, … (*k* − 1) × 2*π*/*k*.

Power and type I error rates of the unmodified Rayleigh test were evaluated over 100,000 stochastic simulations. Only the unmodified Rayleigh test was available in an R package, and the *rayleigh.test* function in the package *circular* was used for this. For all other tests, the *p* value was evaluated by simulation, as the fraction of 1000 samples drawn from a uniform distribution which produced a test statistic at least as great as the observed one. Test statistics for the various tests were defined as follows: Throughout, we assume *k* equal-sized ordered categories, indexed by *j* = 1, … *k*, with observed values *O*_*j*_ and expected values under the null hypothesis of uniformity *E*_*j*_ *.* The total sample size is defined as *n*, so that each *E*_*j*_ is *n*/*k*, and

1 $$ {\sum}_{j=1}^k{O}_j=n $$

For the modified Watson test introduced by Choulakian et al. (1994), we first define

2 $$ {S}_j={\sum}_{i=1}^j\left({O}_i-{E}_i\right). $$

Then, the test statistic is given by

3 $$ {U}_G^2=\frac{1}{nk}{\sum}_{j=1}^k{\left({S}_j-\frac{1}{k}{\sum}_{i=1}^k{S}_i\right)}^2. $$

For the alternative version proposed by Brown (1994), we first define *p*_*j*_ = *E*_*j*_/*n*, *Y*_*j*_ = 0.5(*O*_*j*_ − *E*_*j*_) when *j* = 1; otherwise,

4 $$ {Y}_j={\sum}_{i=1}^{j=1}\left({O}_i-{E}_i\right)+0.5\left({O}_j-{E}_j\right). $$

Then, the test statistic is given by

5 $$ {U}_d^2=\frac{1}{n}\left({\sum}_{j=1}^k{p}_j{Y}_j^2-{\left({\sum}_{j=1}^k{p}_j{Y}_j\right)}^2\right)+\frac{1}{6}{\sum}_{j=1}^k{p}_j^2\left(1-0.5{p}_j\right)+\frac{1}{12n}{\sum}_{j=1}^k{p}_j{\left({O}_j-{E}_j\right)}^2. $$

The method of Freedman (1979) first involves defining

6 $$ {F}_j={\sum}_{i=1}^j\frac{E_i}{n}\kern0.5em {F}_j^n={\sum}_{i=1}^j\frac{o_i}{n}. $$

Then, the test statistic is found from the maximum and minimum values of the differences between these two series, specifically

7 $$ {V}_N=\mathit{\max}\left({F}^N-F\right)+\left|\mathit{\min}\left({F}^N-F\right)\right| $$

For continuous data, the test statistic for the Rayleigh test is the mean vector length *r*. For *n* measurements *M*_*1*_, … *M*_*n*_ recorded as radians on in the interval [0, 2*π*], this is defined as

8 $$ r=\frac{1}{n}\left({\left({\sum}_{i=1}^n\mathit{\sin}\left({M}_i\right)\right)}^2+{\left({\sum}_{i=1}^n\mathit{\cos}\left({M}_i\right)\right)}^2\right). $$

If, as in our case, data has been grouped to take one of *k* discrete values, then, Batschelet (1981) recommends multiplying the test statistic *r* in the Rayleigh test by a correction factor *c*:

9 $$ c=\frac{\frac{\pi }{k}}{\mathit{\sin}\left(\frac{\pi }{k}\right)}. $$

## Results

**Fig. 1:**
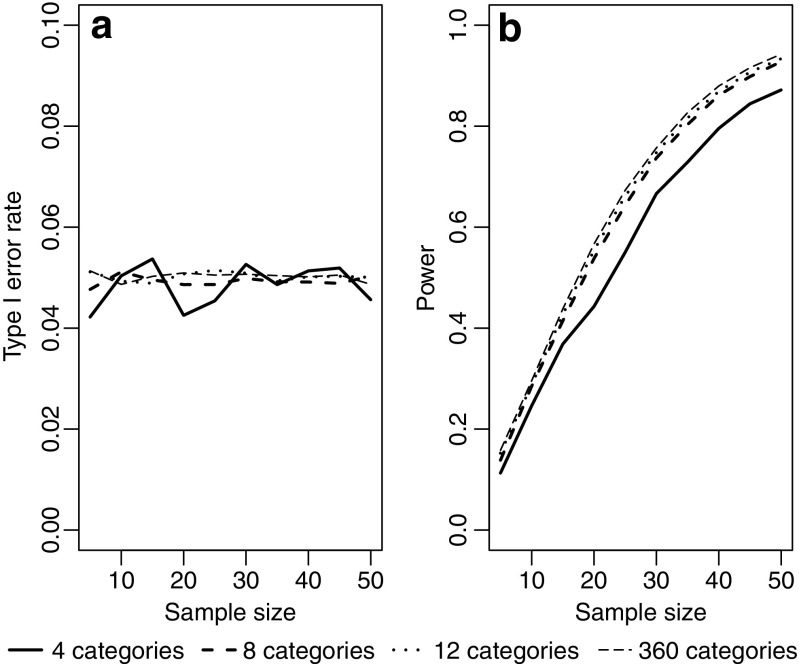
Exploration of the performance of the Rayleigh test on grouped data (drawn from a continuous circular distribution then truncated into either 360, 12, 8 or 4 values representing equal-sized categories). Sample sizes of 5, 10, … 50 were considered. For each combination of the number of categories and sample size, estimates were based on 100,000 samples. **a** The type I error rate when the underlying distribution was uniform. **b** The statistical power when the underlying distribution was von Mises with concentration parameter *κ* = 0.8

**Fig. 2:**
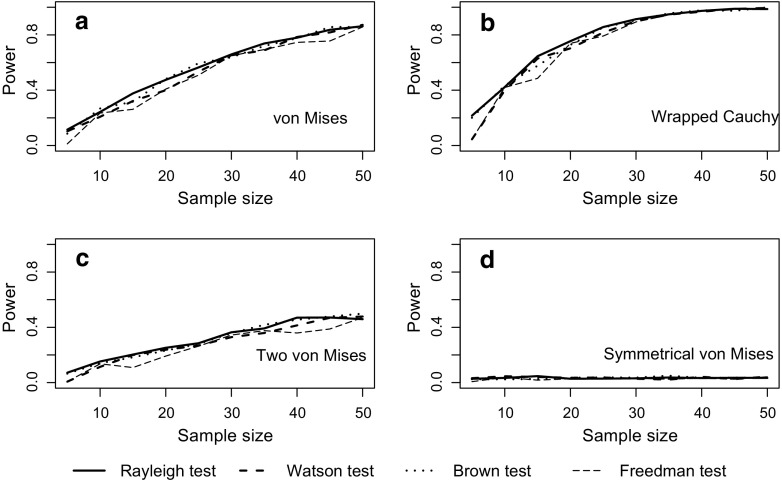
The statistical power of the four tests defined in the “Methods” section for detecting departures from uniformity in circular data grouped into four categories for sample sizes 5, 10, … 50 drawn from four different types of distribution described in full in the “Methods” section. Estimates were based on 1000 replicate samples

**Fig. 3:**
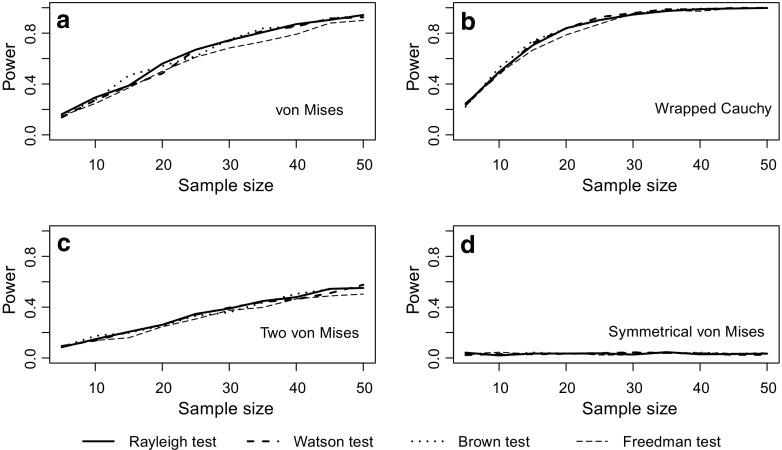
The statistical power of the four tests defined in the “Methods” section for detecting departures from uniformity in circular data grouped into 12 categories for sample sizes 5, 10, … 50 drawn from four different types of distribution described in full in the “Methods” section. Estimates were based on 1000 replicate samples

**Fig. 4:**
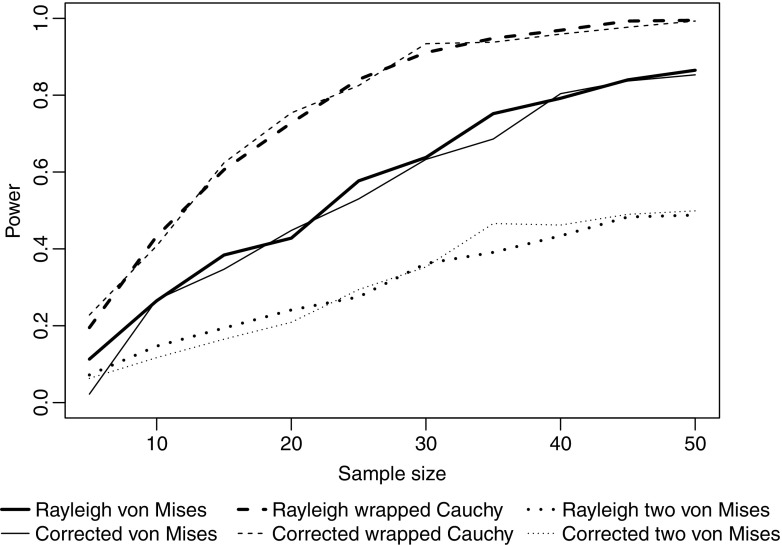
The statistical power of the standard Rayleigh test and the test with the correction factor for grouping recommended by Batschelet (1981) defined in the “Methods” section for detecting departures from uniformity in circular data grouped into four categories for sample sizes 5, 10, … 50 drawn from three different types of distribution described in full in the “Methods” section. Estimates were based on 1000 replicate samples

The Rayleigh test appears to maintain type I error rate at close to the nominal 5% level regardless of how strongly grouped the data are (Fig. 1a). Similarly, although grouping the data leads to a reduction in power, this effect is relatively slight (Fig. 1b). Turning to comparison with the three non-parametric tests designed for grouped data, Fig. 2 shows that there is relatively little difference in power between all four tests, for a broad range of sample sizes and for different types of underlying distribution. All four tests show a good ability to detect unimodal departures from uniformity; their power is reduced for potentially bimodal distributions and is very low indeed if the departure involves two symmetrical distributions at opposite points on the circle. Figure 2 uses extremely grouped data, organised into only 4 categories, but changing to 12 categories makes very little difference (see Fig. 3). In supplementary Figs. S1 and S2, we present analogous results for two further shapes of underlying distribution: a symmetric Jones-Pewsey distribution with parameters chosen to give a smooth broad distribution and an additional skewed version of the symmetric Jones-Pewsey distribution (the sine-skewed Jones-Pewsey). Using the correction suggested by Batschelet (1981) does not seem to offer any significant improvement to the power of the Rayleigh test, even in the extreme case of grouping data into four categories (see Fig. 4). In Fig. S3, we again demonstrate essentially similar observations for two additional distributions.

## Examples

Brown (1994) provides data on the number of marriages in a rural district of Tasmania over the period 1838–1849. For four quarters of the year, there are 16, 25, 22 and 37, respectively. Applying the range of tests considered here, the *p* values associated with the null hypothesis of uniformity between the quarters are 0.17 for the Rayleigh test, 0.06 for Watson’s test, 0.24 for Brown’s test, 0.06 for Freedman’s test and 0.16 for the corrected Rayleigh test. Thus, although there is considerable variation in the *p* values, all tests give no grounds for rejecting the null hypothesis of uniform frequency of marriage across the four quarters, using the criterion of *α* = 0.05.

Bell (1983) recorded the distribution of house martin (*Delichon urbica*) nests in relation to the compass aspect of the wall of the dwelling they were attached to. These were grouped into eight quadrants (N, NE, E, SE, S, SW, W, NW). The observed counts were N = 36, NE = 53, E = 38, SE = 31, S = 26, SW = 21, W = 13, and NW = 35. From visual inspection, it appears that the birds show a preference for NE and E and against SW and W. Testing the hypothesis of no-preference (uniformity) yielded *p*values of < 0.001 for the Rayleigh test, Watson’s test, Brown’s test, Freedman’s test and for the corrected Rayleigh test. Using the criterion of *α* = 0.05, all tests suggest rejection of the null hypothesis of uniform frequency of nest distribution across the eight quadrants of compass aspect. Full code for this example is given in the supplementary material.

## Discussion

### Future developments and broader implications

We can envisage some useful extensions to the results presented here. We have only explored a limited number of different underlying circular distributions here. The Rayleigh test is the most powerful invariant test for continuous data from a von Mises distribution (e.g. Mardia and Jupp 2000). Whilst no longer guaranteed to be optimal, it is known to offer reasonable performance when applied to many other unimodal distributions (Bogdan et al. 2002 and references therein), a conclusion further supported in our simulations. Further, our mixed distribution covered both asymmetric and bimodal distributions. In these situations, the power of the Rayleigh test was reduced compared to symmetric unimodal distributions, but this performance still compared well to alternative tests. Finally, we explored the situation of symmetrical distributions where for continuous data, the Rayleigh test is known to perform poorly (Bogdan et al. 2002). Here, all the tests we considered had similarly very low power. Despite this broad array of underlying distributions, it is possible to imagine alternatives that we have not considered. The most obvious additional distribution to consider is the sinusoid, but a range of other circular distributions is available (see Pewsey et al. 2013 for an overview). Even for the distributions used here, different parameter values could be explored. Lastly, we have only considered equal-sized categories, but there are circumstances (e.g. months of the year) where researchers may be interested in unequal-sized categories. We see no reason to expect that these situations will generate significantly different patterns from those seen here, but such explorations would still be valuable. Pending such investigations, we consider that our work here is a considerable step towards offering hitherto missing empirical support for the widespread use of the Rayleigh test to grouped circular data. Ley and Verdebout (2017) provide the most up-to-date summary of recent research on this test when applied to ungrouped data.

Another logical extension to the investigations here would be extension to another commonly explored situation in circular statistics—investigating the support for two or more samples which have been drawn from the same underlying distribution. For example, researchers might want to test whether there are any differences in seasonal variation of whale strandings on the eastern and western Atlantic coastlines. If the data here is grouped (e.g. into 12 months), then, Pewsey et al. (2013) recommend comparing via a chi-squared approach to a contingency table. However, since this test takes no account of the ordering of months, it may be fruitful to explore if more powerful alternatives for the use of grouped data in this context can be developed.

### Advice to researchers stemming from our study

Here, we found that grouping data into a discrete number of same-sized categories had little effect on either the type I error rate or the power of the Rayleigh test, even for as few as four categories. Further, the power of the Rayleigh test was very similar to that of recommended goodness-of-fit tests designed especially for grouped circular data. On this basis, the application of the Rayleigh test to grouped data might sometimes be appropriate. However, our exploration of grouped data echoes the well-known observation of continuous data that the Rayleigh test can have reduced power when the underlying distribution has multiple peaks. Further, if these peaks are regularly spaced around the circle, then, this reduction in power can be very substantial. Similar loss of power can be seen for the alternative tests for grouped data. Thus, researchers should visually inspect their data before interpreting the outcome of any formal test of the null hypothesis of uniformity. Such inspection could inform both the situation where the null is rejected and the situation where it is not rejected. In the latter case, failure to reject may be related to the issue of low power against some distributions just discussed. As our simulations demonstrate, grouping data leads to a reduction in power, so there are certainly no circumstances where the Rayleigh test can be recommended for grouped data when it would not be recommended for analogous ungrouped data. There are circumstances where the Rayleigh test (and all the tests considered here) has very low power. If researchers have reason to suspect that they are in such a situation, then, they should not apply any of these tests or at the very least treat the results with caution (see Button et al. 2013 for a general discussion of the problems associated with low power).

We have not seen a previous exploration of the grouping correction for the Rayleigh test recommended by Batschelet (1981), although he states (p. 38) that it has minimal effect for *k* > 12. In fact, we find that it has very little effect on the power of the Rayleigh test, even if *k* = 4, so we do not recommend the use of this correction factor when applying the Rayleigh test.

Our suggestion that the Rayleigh test could sometimes appropriately be applied to grouped data is based on its empirical performance in our simulations, ease of use and familiarity; it does not have a theoretical justification. For those researchers who would rather use a method for which there is theoretical justification (rather than just the simulation support provided here), we can offer comparison between three commonly recommended methods based on the existing literature and our investigations. There has been relatively little previous comparison of tests for patterns in grouped circular data. Reijneveld (1990) found that the test of Freedman (1979) gave better or equivalent power to detect sinusoidal deviations from uniformity, compared to a number of tests specifically designed to detect such deviations, and warned that these other tests are unreliable if there is a deviation from uniformity, but it is not sinusoidal. Similar conclusions had been reached previously by Marrero (1983). Here, we found very strong similarity between the three methods tested. None of these methods is currently implemented in software packages that we know of; however, an R code for each of them is provided in the context of the house martin nest distribution example outlined above (see supplementary material). In terms of ease of calculation and speed of implementation, there is little to choose between the three methods. The calculations of Brown’s test are more involved than those of Watson’s but offer no clear performance benefit in our simulations. Freedman’s test offers conceptual similarity to the Kolmogorov-Smirnov test, the modified Watson’s test and the range of other tests commonly used in circular statistics originally developed by Watson. Pending further research, selection can be based on which of these similarities resonates most with the individual researcher.

## Electronic supplementary material

ESM 1 (DOCX 312 kb)

ESM 2 (DOCX 17 kb)
