# Supplementary material for: Consequences of grouped data for testing for departure from circular uniformity
Source: Behav Ecol Sociobiol. 2017 Oct 28;71(11):167. doi: 10.1007/s00265-017-2393-2 (PMC5660840; doi:10.1007/s00265-017-2393-2)
Supplement: Supplementary file 1 — (DOCX 312 kb) [file 265_2017_2393_MOESM1_ESM.docx]

**Consequences of grouped data for testing for departure from circular uniformity**

Behavioral Ecology and Sociobiology

Rosalind K Humphreys and Graeme D Ruxton

School of Biology, University of St Andrews, St Andrews KY16 9TH, UK

Email: rosalindkh08@gmail.com

**Supplement A: Further results for two additional underlying distribution shapes**

**Fig. S1:**


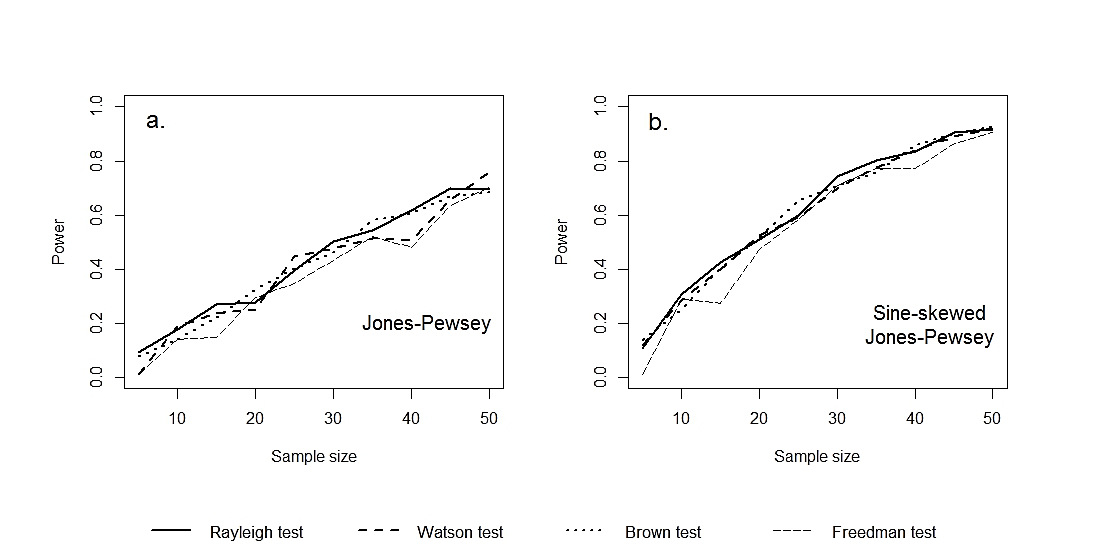


**Figure S1:** Analogous to Fig. 2, we present the statistical power of the four tests defined in the Methods for detecting departures from uniformity in circular data grouped into four categories for sample sizes 5, 10, …..,50 drawn from two additional types of distribution. The Jones-Pewsey function is described fully in Pewsey et al. (2013). We use concentration parameter k = 0.8 and shape parameter f = 3/2 to give a smooth, more gentle peak than our other distributions. The asymmetric sine-skewed Jones-Pewsey function is again described in Pewsey et al. (2013). We use the same parameters as for the symmetric Jones-Pewsey distribution with the addition of a skewness parameter l = p/2 giving a relatively strong asymmetry. Estimates were based on 1000 replicate samples

**Fig. S2:**


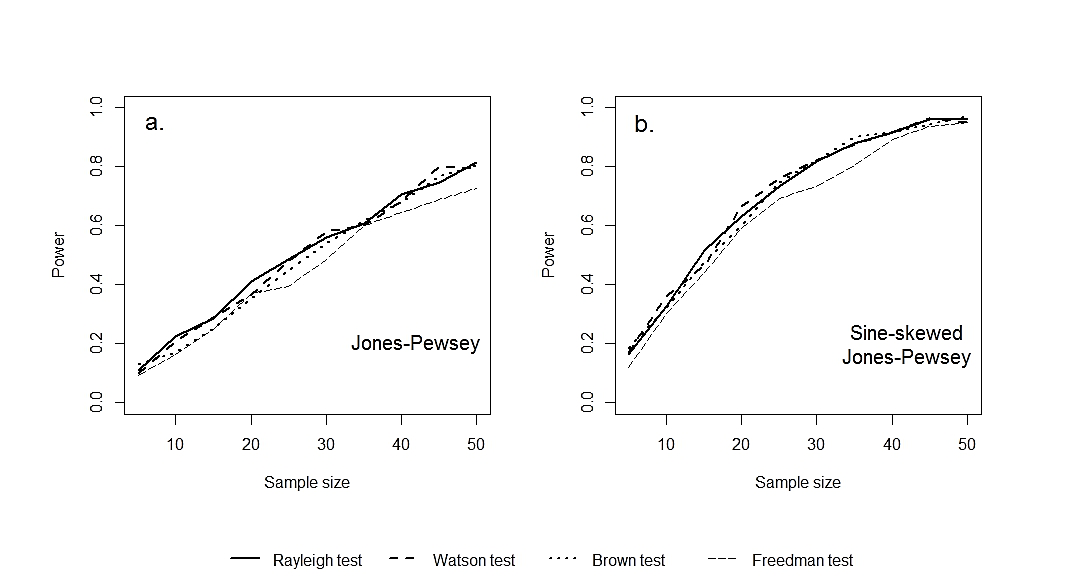


**Figure S2:** As Fig. S1 but with 12 categories rather than 4 (analogous to Fig. 3)


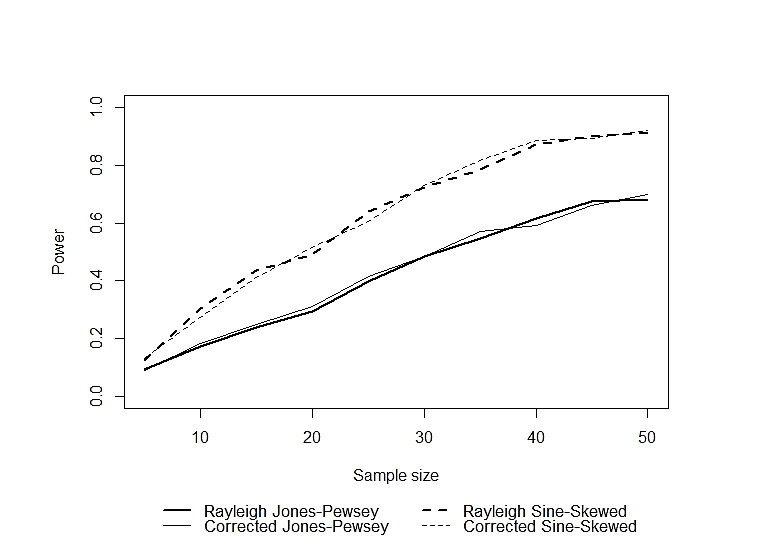
**Fig. S3:**

**Figure S3:** The analogue of Fig. 4 for the two additional distributions described in the caption to Fig. S1
