# Supplementary material for: Consequences of grouped data for testing for departure from circular uniformity
Source: Behav Ecol Sociobiol. 2017 Oct 28;71(11):167. doi: 10.1007/s00265-017-2393-2 (PMC5660840; doi:10.1007/s00265-017-2393-2)
Supplement: Supplementary file 2 — (DOCX 17 kb) [file 265_2017_2393_MOESM2_ESM.docx]

**Consequences of grouped data for testing for departure from circular uniformity**

Behavioral Ecology and Sociobiology

Rosalind K Humphreys and Graeme D Ruxton

School of Biology, University of St Andrews, St Andrews KY16 9TH, UK

Email: rosalindkh08@gmail.com

**Supplement B: R code for the House martin nest distribution example outlined in the ‘Examples’ section**

The code utilised for each test is signposted, including the full details of the tests for which methods are not, to our knowledge, currently implemented in software packages.

library("circular")

##### House martin nest data:

nests <- c(36,53,38,31,26,21,13,35)

allnests <- c(rep(0,36), rep(pi/4, 53), rep(pi/2,38), rep ((3*pi/4),31),rep (pi,26), rep ((5*pi/4),21), rep ((3*pi/2),13), rep ((7*pi/4),35))

##### Rayleigh test (unmodified):

rayleigh.test(allnests)

##### Watson's test, modified due to Choulakian et al. (1994)

#Define Sj calculation function:

sj_calc <- function(observed){

n <- sum(observed)

m <- length(observed)

expected <- c(rep((n/m), times = m))

dif <- observed - expected

sj <- rep(dif[1],m)

for(i in 2:m){

sj[i] = sj[i-1]+dif[i]}

return(sj)}

#Define U2 test statistic calculation function:

U2calc <- function(Sj,N){

k <- length(Sj)

meanSj <- sum(Sj)/length(Sj)

dif_Sj <- (Sj - meanSj)^2

U2 <- (1/(N*k))*sum(dif_Sj)

return(U2)}

#Generate 1000 test statistics from samples drawn from a uniform distribution, with the correct number of samples (n) and categories (m):

n <- 253

m <- 8

univals <- 1000

testset5<- rep(0,univals)

for (f in 1:univals){

data1 <- matrix(rcircularuniform(n, control.circular=list(units="radians")))

data2 <- matrix(data1*(m/(2*pi)))

data3 <- matrix(trunc(data2))

data4 <- matrix(data3*((2*pi)/m))

data5 <- matrix(rep(0,m))

for(k in 1: n){

q <- 1+data3[k]; data5[q] <- data5[q] +1}

sjvals <- sj_calc(data5)

testset5[f] <- U2calc(sjvals,n)}

#Calculate test statistic for the House martin nest data:

sjvals <- sj_calc(nests)

U2test <- U2calc(sjvals,n)

#Calculate p-value for the House martin nest data:

counter <- 0

for(j in 1:univals){

if(testset5[j]>=U2test){counter <- counter+1}}

p <- counter/univals

p

##### Brown's (1994) modification of the Watson test:

#Define Yj calculation function:

Yj_calc <- function(observed){

n <- sum(observed)

m <- length(observed)

expected <- c(rep((n/m), times = m))

dif <- observed - expected

Yj <- 0.5*dif

for(j in 2:m){

for (i in 1:(j-1)){

Yj[j] <- Yj[j]+dif[i]}}

return(Yj)}

#Define U2 test statistic calculation function:

U2calc <- function(Yj,observed){

n <- sum(observed)

m <- length(observed)

expected <- c(rep((n/m), times = m))

dif <- observed - expected

p <- c(rep((1/m), times = m))

temp1 <- 0

for (j in 1:m){

temp1 <- temp1 + (p[j]*(Yj[j]*Yj[j]))}

temp2 <- 0

for (j in 1:m){

temp2 <- temp2 + (p[j]*Yj[j])}

temp2 <- temp2*temp2

temp3 <- 0

for (j in 1:m){

temp3 <- temp3 + ((p[j]*p[j])*(1-(p[j]/2)))}

temp3 <- temp3/6

temp4 <- 0

for (j in 1:m){

temp4 <- temp4 + (p[j]*((observed[j]-expected[j])*(observed[j]-expected[j])))}

temp4 <- temp4/(12*n)

U2 <- ((temp1-temp2)/n)+temp3+temp4

return(U2)}

#Generate 1000 test statistics from samples drawn from a uniform distribution, with the correct number of samples (n) and categories (m):

n <- 253

m <- 8

univals <- 1000

testset5<- rep(0,univals)

for (f in 1:univals){

data1 <- matrix(rcircularuniform(n, control.circular=list(units="radians")))

data2 <- matrix(data1*(m/(2*pi)))

data3 <- matrix(trunc(data2))

data4 <- matrix(data3*((2*pi)/m))

data5 <- matrix(rep(0,m))

for(k in 1: n){

q <- 1+data3[k]; data5[q] <- data5[q] +1}

Yjvals <- Yj_calc(data5)

testset5[f] <- U2calc(Yjvals,data5)}

#Calculate test statistic for the House martin nest data:

Yjvals <- Yj_calc(nests)

U2test <- U2calc(Yjvals,data5)

#Calculate p-value for the House martin nest data:

counter <- 0

for(j in 1:univals){

if(testset5[j]>=U2test){counter <- counter+1}}

p <- counter/univals

p

##### Freedman's (1979) modification of the Kolmogorov-Smirnov test

#Define Vn test statistic calculation function:

Vncalc <- function(observed){

n <- sum(observed)

m <- length(observed)

expected <- c(rep((n/m), times = m))

F <- c(rep((expected[1]/n), times = m))

Fn <- c(rep((observed[1]/n), times = m))

for(i in 2:m){

F[i] = F[i-1]+(expected[i]/n)

Fn[i] = Fn[i-1]+(observed[i]/n)}

dif = Fn - F

Vn = max(dif) + abs(min(dif))

return(Vn)}

#Generate 1000 test statistics from samples drawn from a uniform distribution, with the correct number of samples (n) and categories (m):

n <- 253

m <- 8

univals <- 1000

testset5<- rep(0,univals)

for (f in 1:univals){

data1 <- matrix(rcircularuniform(n, control.circular=list(units="radians")))

data2 <- matrix(data1*(m/(2*pi)))

data3 <- matrix(trunc(data2))

data4 <- matrix(data3*((2*pi)/m))

data5 <- matrix(rep(0,m))

for(k in 1: n){

q <- 1+data3[k]; data5[q] <- data5[q] +1}

testset5[f] <- Vncalc(data5)}

#Calculate test statistic for the House martin nest data:

Vntest <- Vncalc(nests)

#Calculate p-value for the House martin nest data:

counter <- 0

for(j in 1:univals){

if(testset5[j]>=Vntest){counter <- counter+1}}

p <- counter/univals

p

##### Corrected Rayleigh's test (Batschelet, 1981)

#Define r calculation function:

r_calc <- function(x){

lenx <- length(x)

cosdata<-sum(cos(x))

sindata<-sum(sin(x))

r <- ((sindata*sindata)+(cosdata*cosdata))/lenx

return(r)}

#Define rc test statistic calculation function:

rc_calc<-function(r,m) {

lambda<-(2*pi)/m

c<-((lambda/2)/(sin(lambda/2)))

rc<-r*c

return(rc)}

#Generate 1000 test statistics from samples drawn from a uniform distribution, with the correct number of samples (n) and categories (m):

n <- 253

m <- 8

univals <- 1000

testset5<- rep(0,univals)

for (f in 1:univals){

data1 <- matrix(rcircularuniform(n, control.circular=list(units="radians")))

data2 <- matrix(data1*(m/(2*pi)))

data3 <- matrix(trunc(data2))

data4 <- matrix(data3*((2*pi)/m))

rvals <- r_calc(data4)

testset5[f] <- rc_calc(rvals,m)}

#Calculate test statistic for the House martin nest data:

rvals <- r_calc(allnests)

correctiontest <- rc_calc(rvals,m)

#Calculate p-value for the House martin nest data:

counter <- 0

for(j in 1:univals){

if(testset5[j]>=correctiontest){counter <- counter+1}}

p <- counter/univals

p
